# Young Children's Comprehension of Temporal Relations in Complex Sentences: The Influence of Memory on Performance

**DOI:** 10.1111/cdev.12412

**Published:** 2015-08-26

**Authors:** Liam P. Blything, Robert Davies, Kate Cain

**Affiliations:** ^1^ Lancaster University

## Abstract

The present study investigated 3‐ to 7‐year‐olds' (*N *=* *91) comprehension of two‐clause sentences containing the temporal connectives *before* or *after*. The youngest children used an order of mention strategy to interpret the relation between clauses: They were more accurate when the presentation order matched the chronological order of events: “He ate his lunch, before he played in the garden” (chronological) versus “Before he played in the garden, he ate his lunch” (reverse). Between 4 and 6 years, performance was influenced by a combination of factors that influenced processing load: connective type and presentation order. An independent measure of working memory was predictive of performance. The study concludes that the memory demands of some sentence structures limits young children's comprehension of sentences containing temporal connectives.

Successful comprehension results in an integrated and coherent mental representation of the state of affairs described in a text, rather than a verbatim record of the specific words or syntactic structures (Johnson‐Laird, 1983; Van Dijk & Kintsch, 1983). Critically, adult readers and listeners encode the relations between events on several dimensions, including temporality, the order in which events occur (Gennari, 2004; Zwaan, 2008; Zwaan & Radvansky, 1998). Temporal connectives such as *before* and *after* are one source of linguistic information that specifies the order of events, and as a result, they aid the comprehension of multiple‐clause sentences and the construction of an accurate and coherent meaning‐based representation (Costermans & Fayol, 1997). Although temporal connectives are produced in children's speech from around 3 years of age (Diessel, 2004), children have difficulty on tasks designed to assess the comprehension of these connectives up to at least 12 years of age (Pyykkönen & Järvikivi, 2012). That is, young school‐aged children produce temporal connectives before they can comprehend them in spoken language.

In this research, we focus on the development of comprehension of sentences containing the temporal connectives *before* and *after* in 3‐ to 7‐year‐olds. Our findings indicate the age at which competence emerges in the use of connectives, and how this is related to different sentence structures. Our observations advance understanding of the development of competence in temporal connectives by revealing the influence of memory skills in the improvements in performance evident during early childhood.

When children do not understand a temporal connective, they can use different strategies to understand and represent the relation between two events in a two‐clause sentence containing a temporal connective, rather than using the precise linguistic information provided by the connective itself (Clark, 1971). Two strategies that we consider are a world knowledge strategy and an order of mention strategy. World knowledge may support correct interpretation of event order when the events typically occur in a set order, for example, “She put on her boots, after she put on her socks.” The order of events in such sentences can be understood without using the information provided by the connective. When there is no typical order for two events, as in, “She put on her hat, after she put on her scarf,” language comprehenders can only interpret the order correctly if they understand the relation signaled by the connective. Between 3 and 5 years of age, children appear to rely on world knowledge, rather than knowledge of the connective: They are better at comprehending the sequence of events expressed in sentences when the sequences are typical, and thus supported by world knowledge, compared to when event order is arbitrary (e.g., French & Brown, 1977; Keller‐Cohen, 1987; Trosborg, 1982).

Children may also construct a correct interpretation of the sequence of events expressed in a sentence by assuming that the event sequence corresponds to the order in which the events were mentioned: an order of mention strategy (Clark, 1971). If young children are using this strategy, they should find it easier to comprehend sentences in which the order of mention corresponds to the order of events, as in chronologically ordered sentences such as, “She put on her hat, before she put on her scarf,” compared to reverse chronological sentences such as, “She put on her scarf, after she put on her hat.” An order of mention strategy will result in an incorrect interpretation of event order in the latter. Between the ages of 3 and 5 years, children perform more accurately on chronological sentences than on reverse chronological sentences (Clark, 1971; French & Brown, 1977; Johnson, 1975; Trosborg, 1982). This finding indicates that young children employ an order of mention strategy to comprehend the temporal order of events in multiple‐clause sentences. Thus, children can resort to two strategies, world knowledge or order of mention, to respond appropriately to connectives without fully understanding them.

These studies inform us that 3‐ to 5‐year‐old children do not have full understanding of the meaning of *before* and *after* and provide us with an insight into the strategies that young children might use to process complex sentences that include a temporal connective. However, as mentioned earlier, even 12‐year‐olds do not perform at adult levels in studies designed to assess the comprehension of sentences containing temporal connectives (Pyykkönen & Järvikivi, 2012). The question we ask is this: What factors drive the comprehension of complex sentences containing temporal connectives once children have developed an appreciation for the meaning of *before* and *after*?

The extant literature suggests that three key factors may influence the comprehension of sentences that include connectives: the relative familiarity of the connective in terms of its frequency of occurrence in a child's linguistic experience, the relation between the order of mention of the connective and the order of events being described by the connective, and the position of the connective in a sentence. Each effect can be explained in relation to the impact of variation in the demands on processing capacity imposed by sentences including connectives. Developmental improvements would be predicted by capacity‐constrained theories of comprehension which propose that comprehenders with low working memory capacity are less likely to retain a full and accurate representation of a sentence during comprehension, particularly when that sentence carries high memory demands (e.g., Just & Carpenter, 1992).

To establish the motivation for our study, we review relevant research. One factor that should be expected to affect comprehension performance is the relative familiarity of different temporal connectives according to the language experience of the child. Clark (1971) found earlier competence for *before* than for *after* in 3‐ to 5‐year‐olds. She attributed this difference in age of acquisition to the semantic features of each term: *before* indicates the prior event, whereas *after* does not, making the latter more semantically complex. Another reason for earlier competence for *before* relative to *after* is differential exposure to these temporal terms. As is evident in large language corpora such as the British National Corpus (Leech, Rayson, & Wilson, 2001), *after* occurs more often than *before* as a preposition or adverb, as in, “The dog chased after the ball,” in addition to its use as a temporal connective. As a result, it may be more difficult for children to activate their knowledge of *after* as a temporal connective compared to *before*.

Another factor that may influence performance is the relation between the order of mention of the connective and the order of events being described by the connective. As noted, children who do not understand the semantics of a temporal connective are more likely to be accurate at comprehending sentences in which the order of mention of events is congruent with the chronological order of occurrence of the events (e.g., Clark, 1971). Importantly, once a competent understanding of the connective itself emerges, a processing difficulty for reverse chronological sentences may persist as a function of high memory load (Ye et al., 2012).

It has long been known that the mental representation of a multiple‐clause sentence encodes its meaning, not specific words or syntactic structures (Bransford, Barclay, & Franks, 1972). For a chronological order sentence, information about the sequence of events specified in two clauses linked by a connective can be assimilated into a congruent meaning representation for the sentence incrementally, as the events are mentioned. In contrast, the comprehender cannot incrementally construct a correct interpretation of the sequence of events for a reverse chronological order sentence such as, “Before she put on her scarf, she put on her hat,” but must wait until the second clause is presented. The greater demands on memory imposed by reverse chronological sentences in this account can be expected to cause comprehension problems for young children. Consistent with this prediction, even adults find sentences with an initiating connective harder to process when the events are presented in reverse chronological order (Münte, Schiltz, & Kutas, 1998; Ye et al., 2012).

The position of the connective in the sentence was not a factor directly manipulated in our study, but we consider it here because it will vary as a function of the connective (before vs. after) and manipulation of order (chronological vs. reverse). Temporal connectives can appear in either a sentence medial position, as in, “She put on her hat, before she put on her scarf,” or a sentence initial position, as in, “Before she put on her scarf, she put on her hat.” In an analysis of children's natural language production, Diessel (2004) found a strong preference for the sentence medial position for temporal connectives in the productions of children aged between 2 and 5 years (see also Diessel, 2008, for similar work with adults). This preference can be explained by noting that if a connective occurs in a sentence medial position, incremental word‐by‐word processing of the sentence meaning is afforded, but that when a connective occurs in the sentence initial position, the comprehender (or producer) cannot simply process (or plan) the sentence word by word. Thus, the position of the connective in the sentence may influence comprehension through the variation in working memory demands that arise through sentence position. When processing sentences that contain connectives in the sentence initial position, the comprehender must maintain the information provided by the connective in memory while processing the event of the first clause, and then use the stored connective information to link the event specified in the first clause correctly with the event specified in the second clause.

When processing a sentence medial connective, the information required to link events specified in the first and second clauses will be available roughly when it is required, reducing the period during which the content of the first clause must be maintained in working memory prior to linkage with the second clause. The assumption is therefore that connectives in the medial position are preferred because they can be processed accurately while making fewer demands on memory. Consistent with this account, studies of older children and adults have indicated the general use of an incremental processing strategy for sentences joined medially by connectives (Cain & Nash, 2011; Traxler, Bybee, & Pickering, 1997). For young children, who have low working memory capacity, a connective appearing in the sentence initial position may therefore be harder to comprehend.

Only one study to date speaks to these three factors in relation to children's (and adults') mental representation. This study by Pyykkönen and Järvikivi (2012) found that for 8‐ to 12‐year‐olds, chronologically ordered sentences that could be processed incrementally (before‐chronological) were easier to comprehend than reverse‐ordered sentences that also had a connective in the medial position but that could not be processed incrementally (after‐reverse). Sentences in which the connective appeared in the initial position (before‐reverse and after‐chronological) were of similar and intermediate difficulty for the children, whereas adults performed at ceiling on all sentence types. Pyykkönen and Järvikivi's study clearly demonstrates the need to consider that differences in sentence position, which will arise through the manipulation of connective and order, might influence the comprehension of sentences with temporal connectives. However, Pyykkönen and Järvikivi's task allowed rereading and reflection on the sentence. For that reason, their findings cannot be interpreted directly in terms of the differing processing demands imposed by sentences with different structures involving temporal connectives. We set out to advance understanding of young children's comprehension of connectives by considering the impact of order, connective type, and position, by using a task that promoted response types that would allow interpretation of effects in terms of memory load.

## The Present Study

Previous research has identified the strategies that very young children might use to process multiple‐clause sentences containing temporal connectives, but has not investigated why these sentences remain hard for children to process for several years after they appear in their spoken language productions. We compared consecutive age groups between 3 and 7 years of age to pinpoint the moment of developmental change. Our aim was to determine when children shift from using strategies such as order of mention or world knowledge to comprehend the chronological order of events in sentences that contain temporal connectives, to using the connective itself as a linguistic device that signals order. Furthermore, we aimed to elucidate the reasons why these sentences are often misunderstood even after children appreciate the different orders signaled by *before* and *after*. We compared comprehension of two‐clause sentences joined by *before* and *after* and manipulated whether the event sequence was presented in chronological or reverse order. In this way, position of connective varied as a function of these two factors. Thus, the design included the following sentence types: before‐chronological order (medial position), before‐reverse order (initial position), after‐chronological order (initial position), and after‐reverse order (medial position). We also manipulated whether the events in the two clauses typically occurred in a set order (world knowledge present) or not (world knowledge absent). The manipulation of world knowledge in conjunction with these other factors allowed us to identify whether children used an order of mention strategy or relied on world knowledge when they did not possess robust working knowledge of the connective.

Our interest in the language processing demands posed by connectives led us to select a task that had low cognitive performance demands. The majority of previous studies examining young children's comprehension of temporal connectives have used an act‐out task, which has high cognitive demands (Ambridge & Rowland, 2013). Here, to capture early competence and to minimize the processing demands, we assessed comprehension with a simple forced‐choice task. Children listened to a two‐clause sentence in which the order of two events was signaled by a connective (*before* or *after*) while viewing an image of each clause on a touch screen monitor. After each sentence, they selected which of the two events happened first. The use of images depicting the events in sentence stimuli reduces memory load (e.g., Vion & Colas, 2005). Previous successful use of touch screen technology for capturing early comprehension competence has been reported with children as young as 18 months (Friend & Keplinger, 2008).

Knowledge of *before* should be acquired earlier than *after* according to both the semantic complexity and frequency of exposure accounts. Therefore, in general, *before* sentences should elicit a greater number of accurate responses than *after* sentences. We hypothesized that the youngest children's pattern of performance would indicate that they did not have robust knowledge of the temporal relation signaled by the connective (consistent with the previous research detailed earlier) and would rely on a strategy using either order of mention or world knowledge to comprehend sentences. Previous research has not identified a preference for either strategy, so we did not make specific predictions on this point. We predicted that the older children would generally perform above chance on both connectives, because they had more secure knowledge of the specific meaning of the connectives.

However, the previous literature discussed earlier motivated us to predict that older children's performance would be affected by the processing demands of different sentence types (e.g., Pyykkönen & Järvikivi, 2012; Ye et al., 2012). Taken together, this literature identifies three key factors that vary the grammatical structure of sentences including connectives, namely: connective type, the order of events, and connective position. This variation may also impact the demands on processing capacity. Consistent with a capacity‐constrained theory of comprehension, we expected that children would perform worse on sentences that inflict high memory demands during clause integration (e.g., Just & Carpenter, 1992). For example, before‐chronological sentences such as, “She put on her hat, before she put on her scarf,” were expected to elicit the most accurate level of performance because the order (chronological) and connective position (medial) combined to allow word‐by‐word incremental processing. In comparison, before‐reverse (initial position, reverse order), after‐chronological (initial position, later acquired connective), and after‐reverse (reverse order, later acquired connective) sentences would elicit less accurate performance because such sentences each carry two features associated with high memory load.

Given the potential explanation of performance patterns in terms of processing load, we included an independent measure of memory in our analysis of comprehension performance to examine if the influence of sentence structure on comprehension would be modulated by children's memory capacities. We predicted that memory would be a significant predictor of performance, in general.

## Method

### Participants

Ninety‐one children aged 3–7 years participated in the study. All were native English speakers from schools and preschools that served mixed socioeconomic catchment areas in the North West region of England. No children had reported language disabilities. Children were in four different school year groups: twenty‐two 3‐ to 4‐year‐olds (age range = 3 years 3 months to 4 years 4 months; 13 boys), twenty‐one 4‐ to 5‐year‐olds (age range = 4 years 5 months to 5 years 5 months; 14 boys), twenty‐four 5‐ to 6‐year‐olds (age range = 5 years 5 months to 6 years 5 months; 11 boys), and twenty‐four 6‐ to 7‐year‐olds (age range = 6 years 5 months to 7 years 4 months; 13 boys). Data collection took place between January and July 2013. Written parental consent was obtained for all children, and children provided oral assent before each session. All children had age‐appropriate receptive language assessed using the British Picture Vocabulary Scales–III (Dunn, Dunn, Styles, & Sewell, 2009). Full details are reported later.

### Materials and Procedure

All children completed three assessments: a connective comprehension task, a measure of memory, and a measure of receptive vocabulary. The connectives task was administered over two separate sessions. Each session lasted no longer than 20 min. One session included the vocabulary assessment, the other the memory assessment.

#### Connective Comprehension Task

Comprehension of *before* and *after* was measured using a touch‐screen paradigm. Sixty‐four 2‐clause sequences were constructed, each representing two events that were related by world knowledge. All sequences referred to one actor and two objects. Each of the 64 items was counterbalanced into one of eight lists. In each list there were 32 sentences assessing eight conditions (shown in Table 1) that resulted from three manipulated factors: presence or absence of world knowledge to support the relation between the two events, the temporal connective (*before* vs. *after*), and the presentation order of events (chronological vs. reverse chronological). The manipulations of connective type and order of events in turn resulted in sentences in which for both *before* and *after* the connective could appear in either initial or medial position. Thus, for *before* sentences, the connective appeared in the medial position (as shown in Table 1) when events were presented in chronological order, and in the sentence initial position when the events were presented in reverse chronological order. The reverse was true for sentences containing *after*.

**Table 1:** Sentence Conditions

	Before		After	
	Chronological	Reverse	Chronological	Reverse
World knowledge present	He poured the ketchup, before he ate the burger	Before he ate the burger, he poured the ketchup	After he poured the ketchup, he ate the burger	He ate the burger, after he poured the ketchup
World knowledge absent	He put on the sandals, before he ate the burger	Before he ate the burger, he put on the sandals	After he put on the sandals, he ate the burger	He ate the burger, after he put on the sandals

**Figure 1:**
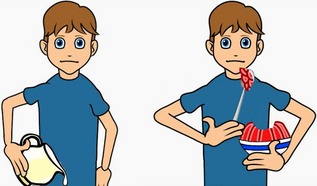
Example presentation of an animation freeze frame (cream, jelly).

For each clause, an animated cartoon was created using Anime Studio Pro 9.1 (Smith Micro Software, 2012). Each cartoon depicted the actor, action, and object of a clause (e.g., Tom pouring a ketchup bottle; Tom eating a hotdog). Each animated segment lasted for 3 s and explicitly encapsulated only the object (e.g., hotdog) from one clause, while the object (e.g., ketchup) from the other clause was not present. Each animation ended with a freeze frame judged by the first and third authors to best represent the action of that clause. An example of freeze frame is provided in Figure 1. Each visual stimulus (e.g., Tom eating a hotdog) was 486 pixels in height and did not exceed the left or right half of the presentation (486 × 872 pixels).

Children first saw the two animations, shown sequentially. The animation on the right‐hand side of the screen was shown first, followed by the animation on the left‐hand side of the screen. Children were instructed: “Listen carefully and touch the thing Tom/Sue did first” (name selected to match gender of child) and the narration of the sentence was played (over headphones). A response window was opened with a short beep and was closed by a blank screen once the child had responded. Both order of appearance and side of presentation for the visual representations of the target and nontarget clauses were counterbalanced across trials.

The experiment was run using the PsyScript 3.2.1 (Slavin, 2013) scripting environment on a Macintosh laptop connected to a touch‐screen monitor with items presented in a random order. Correct responses were recorded as those items for which the child touched the target action that was the first event to occur in the sentence.

Children completed a minimum of four practice trials to ensure that they understood the procedure before the test phase. Practice trial instructions emphasized the importance of making judgments based solely on the narrated sentence, not the visual stimuli. Paired‐sample *t* tests revealed no significant effect of order or side of presentation on accuracy (for all comparisons, *p *>* *.1; data were reflected and log transformed for the two oldest age groups because their data were not normally distributed).

#### Vocabulary

Receptive vocabulary was assessed using the British Picture Vocabulary Scales–III (Dunn et al., 2009) to ensure that the sample had age‐appropriate vocabulary skills. In this task, the child has to point to one of four pictures that best illustrates the meaning of a word spoken aloud by the researcher. Testing is discontinued when a specified number of errors have been made. All children had a standardized score above 85 and the mean scores (± *SD*) indicate that each age group was performing at an age‐appropriate level: 3‐ to 4‐year‐olds = 109.27 (10.37), 4‐ to 5‐year‐olds = 111.76 (6.16), 5‐ to 6‐year‐olds = 105.83 (8.67), and 6‐ to 7‐year‐olds = 103.88 (8.02). Thus, no children were excluded for weak receptive language skills.

#### Memory

Each child completed the digit span task from the Working Memory Battery for Children (Pickering & Gathercole, 2001) to assess memory. In this task, the child hears a string of digits, read out by the researcher, and is then asked to recall the digits in the same order. The easiest level comprises strings of two digits, and the number of items in the string is increased until the child cannot recall all of the digits after three successive attempts. This is the most suitable assessment of memory for our age range, because 4‐year‐olds perform at floor on more complex measures of working memory (Gathercole, Pickering, Ambridge, & Wearing, 2004). Raw scores were used for the analysis. The test–retest reliability reported in the manual for children aged 5–7 years is *r *=* *.81.

#### Design

A 4 × 2 × 2 × 2 mixed design was used. The between‐subjects independent variable was year group (3–4, 4–5, 5–6, and 6–7 years) and the within‐subjects variables were world knowledge (present, absent), connective (*before*,* after*), and order (chronological, reverse chronological). The dependent variable was accuracy.

## Results

A total of 5,824 responses were recorded. Before analysis, data were screened to remove potential distortions from the norm. Three children from the oldest age group were removed (192 responses, 3.3%) because they were identified in by‐age box plots as outliers who were performing at floor level in accuracy. Therefore, 5,632 responses were included. The removal of these participants did not affect the pattern of the reported results.

### Analysis Strategy

A series of generalized linear mixed effects models (GLMMs; Baayen, Davidson, & Bates, 2008; Barr, Levy, Scheepers, & Tilly, 2013) were fitted to the data in the R statistics environment (R Development Core Team, 2014) using the lme4 package (Bates, Maechler, & Bolker, 2014). This method is essentially an extension of logistic regression, such that a GLMM analysis estimates the fixed effects due to experimentally manipulated variables while taking into account random error variance due to differences between participants or between stimulus items sampled for the study. We followed the recommendations of Barr et al. (2013) by estimating fixed effects in models that included random effects terms corresponding to both random differences between participants or items in overall accuracy of responses elicited (random intercepts) and random differences between participants or items in the slopes of the effects of world knowledge, connective, and order condition. As a maximal random effects model did not converge, we used the likelihood ratio test (Barr et al., 2013; Pinheiro & Bates, 2000) to test whether the inclusion of fixed or random effects was warranted by superior model fit to data. That is, we added as many slopes as were found to be warranted. Each of the final models incorporated random intercepts for both participants and item effects, and by‐participant random slopes for both connective and order effects.

The raw memory scores (*M *± *SD*) demonstrated age‐related improvements: 3‐ to 4‐year‐olds = 15.36 (3.50), 4‐ to 5‐year‐olds = 19.67 (2.94), 5‐ to 6‐year‐olds = 22.67 (4.02), and 6‐ to 7‐year‐olds = 25.42 (4.48). In addition, the standardized scores of memory were within the normal range of 85–115 for each age group: 4‐ to 5‐year‐olds = 91.90 (10.35), 5‐ to 6‐year‐olds = 97.75 (15.15), and 6‐ to 7‐year‐olds = 100.96 (20.42). Standardized scores are not provided for 3‐ to 4‐year‐olds.

In the following, we first describe the optimum model for the full data set, with age, order, and connective entered as fixed effects (Model 1, Table 2). We then further examined the significant interaction between age, connective, and order, found in the full data set model, by conducting simple interaction analyses of the effects of connective and order for each group separately. Table 3 presents the analysis with different age groups to determine their use of comprehension strategies. Finally, in Table 4 (Model 2), we returned to our analysis of the full data set to examine whether a model with memory included as a fixed effect fitted the data better than a model without. In each analysis, world knowledge had no significant main effects nor any significant interactions (all *p*s > .14), and the fit of the model was improved upon its removal, χ^2^(8) = 22.53, *p *<* *.01. Therefore, following recommendations for obtaining an optimal model by Barr et al. (2013), the effect of world knowledge is not included in the models that we present.

**Table 2:** Summary of Generalized Linear Mixed Effects Model for the Log Odds of Accuracy Responses: Effects and Interactions of Age, Order, and Connective

Fixed effects	Estimated coefficient (*b*)	*SE*	*t*	*p*(>|*z*|)
Intercept	−2.42	0.66	−3.67	.01
Age	**0.06**	**0.01**	**5.62**	**< .01**
Order	1.11	0.61	1.81	.07
Connective	−0.63	0.73	−0.86	.39
Order × Connective	−1.16	0.69	−1.67	.09
Age × Order	−0.01	0.01	−1.47	.14
Age × Connective	0.02	0.01	1.44	.15
Age × Order × Connective	**0.03**	**0.01**	**2.19**	**.03**

Note

Fixed‐effects labels: Age = effect of age (in months); Order = effect of order, chronological (reference level) versus reverse chronological; Connective = effect of connective, before (reference level) versus after. Boldface indicates the predictor is significant at *p *< .05 or better.

**Table 3:** Summary of Generalized Linear Mixed Effects Models (per Age Group) for the Log Odds of Accuracy Responses: Effects and Interactions of Order and Connective

	Ages 3–4				Ages 4–5				Ages 5–6				Ages 6–7			
	(*b*)	*SE*	*z*	*p*	(*b*)	*SE*	*z*	*p*	(*b*)	*SE*	*z*	*p*	(*b*)	*SE*	*z*	*p*
Full models																
(Intercept)	0.17	0.22	0.76	.44	0.78	0.33	2.39	.02	1.52	0.33	4.65	< .01	3.18	0.37	8.64	< .01
Order	**0.56**	**0.26**	**2.18**	**.03**	0.36	0.37	0.97	.33	0.43	0.29	1.46	.15	−0.67	0.37	−1.78	.08
Connective	0.28	0.19	1.45	.15	0.10	0.39	0.26	.80	**0.73**	**0.34**	**2.18**	**.03**	0.70	0.40	1.74	.08
Order × Connective	−0.41	0.28	−1.45	.15	**0.81**	**0.36**	**2.27**	**.02**	**2.95**	**0.81**	**3.66**	**< .01**	**2.16**	**0.89**	**2.44**	**< .01**
Simple‐effects models																
Before models																
(Intercept)	—	—	—	—	0.86	0.33	2.65	.01	2.20	0.25	8.68	< .01	3.79	0.49	7.81	< .01
Order	—	—	—	—	**1.11**	**0.30**	**3.72**	**< .01**	**3.75**	**0.77**	**4.88**	**< .01**	1.69	0.91	1.85	.06
After models																
(Intercept)	—	—	—	—	0.94	0.39	2.43	.02	1.54	0.33	4.65	< .01	2.87	0.33	8.62	< .01
Order	—	—	—	—	0.17	0.38	0.45	.65	0.25	0.21	1.18	.24	−0.50	0.28	−1.82	.07

Note

Fixed‐effects labels: Age = effect of age (in months); Order = effect of order, chronological (reference level) versus reverse chronological; Connective = effect of connective, before (reference level) versus after. Boldface indicates predictor is significant at *p *< .05 or better.

**Table 4:** Summary of Generalized Linear Mixed Effects Model for the Log Odds of Accuracy Responses: Effects and Interactions of Memory, Age, Order, and Connective

Main model	*M* (*b*)	*SE*	*t*	*p*(> |*z*|)
(Intercept)	−2.77	0.66	−4.23	.00
Age	**0.03**	**0.01**	**2.10**	**.04**
Memory	**0.10**	**0.04**	**2.68**	**< .01**
Order	1.11	0.63	1.77	.08
Connective	−0.42	0.74	−0.57	.57
Order × Connective	**−1.44**	**0.71**	**−2.04**	**.04**
Age × Order	−0.01	0.01	−0.98	.33
Age × Connective	**0.03**	**0.02**	**2.09**	**.04**
Memory × Order	0.00	0.04	−0.08	.94
Memory × Connective	−0.07	0.04	−1.46	.14
Age × Order × Connective	0.00	0.02	0.13	.89
Memory × Order × Connective	**0.09**	**0.05**	**1.86**	**.06**

Note

Fixed‐effects labels: Age = effect of age (in age); Order = effect of order, chronological (reference level) versus reverse chronological; Connective = effect of connective, before (reference level) versus after. Boldface indicates predictor is significant at *p *< .05 or better.

The inferential statistics for each model are presented in Tables 2, 3, 4, respectively. These summarize the main effects and interactions of age, order, and connective. The first column provides the coefficient estimates of effects (*b*) due to experimental conditions, which is the change in the log odds of accuracy response associated with each fixed effect. A positive coefficient indicates that the effect of differences between conditions was to increase the odds that a response would be correct, while a negative coefficient indicates that the effect of a factor was to decrease the odds that a response would be correct.

### Main Analysis

**Figure 2:**
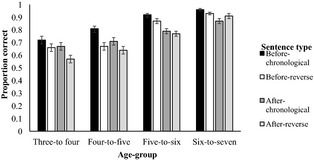
Mean proportion correct (with standard error bars) for each experimental condition by age group.

Model 1 (Table 2) shows that accuracy of response was significantly affected by participant age, indicating a developmental improvement in accuracy from 3 to 7 years. In general, chronological sentences were comprehended as well as reverse chronological sentences. Similarly, there was no difference between accuracy for *before* and *after* sentences. Order and connective effects did not interact with each other, or by age. There was a significant three‐way interaction between age, order, and connective. Figure 2 shows the mean accuracy scores for all observations to each experimental condition (collapsed over world knowledge).

Given the significant interaction between the effects of age, order, and connective conditions, we conducted simple interaction analyses to examine the effects of order and connective on the responses for each age group considered separately. These are reported next and summarized in Table 3.

### Analyses of Individual Age Groups

A main effect of order, only, was found in the analysis of the 3‐ to 4‐year‐olds' data, because this youngest age group comprehended chronological sentences more accurately than reverse chronological sentences. There was no main effect of connective for the two youngest age groups, indicating that they comprehended *before* and *after* equally poorly. In contrast, there was a main effect of connective in the analysis of the data from the 5‐ to 6‐year‐olds and a similar, but nonsignificant, effect in the analysis of the 6‐ to 7‐year‐olds' data. The interaction between order and connective was not significant for the youngest age group. In contrast, this interaction was significant for each of the three oldest age groups.

The order by connective interaction for each of the three older age groups was explored further by examining performance for the *before* and *after* items separately (see also Figure 2). The two middle age groups displayed a main effect of order with *before* items, but not with *after* items. That is, before‐chronological sentences were comprehended better than before‐reverse sentences, whereas after‐chronological sentences were comprehended as well as after‐reverse sentences. Therefore, 4‐ to 6‐year‐olds displayed a significant preference for *before* sentences that were presented in chronological order. These effects were not significant for the oldest age group, for whom performance was much higher in general.

Model 2 (Table 4) indicates that when memory is incorporated in the model of the full data set, it predicts performance over and above age. Indeed, this model was a significantly better fit to the data than when the same model was run without memory, χ²(4) = 13.93, *p *<* *.01. There was a significant two‐way interaction between order and connective. Of particular note, the three‐way interaction between age, order, and connective was no longer significant but, instead, the three‐way interaction between memory, order, and connective neared significance (*p *=* *.06). This latter finding provided converging evidence that age effects were partly a proxy for memory, a conclusion corroborated by the strong correlation between these two variables (*r *=* *.71).

Of course, an alternative explanation of any memory effects in developmental studies is variation in long‐term knowledge of language (e.g., Kidd, 2013; Klem et al., 2015). To test this account, Model 3 tested the same three factors as Model 2 (age; order of events; connective) but with the receptive vocabulary scores included instead of performance on the assessment of memory. This model (Model 3) was not a significantly better fit to the data than Model 1, χ²(4) = 8.82, *p *<* *.07. A final model (Model 4) was also tested that included the same factors as Model 1 and with both memory and vocabulary included. While this model was a significantly better fit than the Model 1, χ²(8) = 20.02, *p *=* *.01, it did not significantly improve the fit compared to Model 2, which included just memory, χ²(4) = 6.07, *p *=* *.19. Together, these comparisons between models indicate that memory, not vocabulary, is driving performance on our sentence comprehension task. For these reasons, we do not include the output for either of the models that incorporated vocabulary (Models 3 and 4; see Data S1, available from the contact author, for this information).

## Discussion

The aim of the present study was to identify the age at which children accurately use *before* and *after* to understand the temporal relation between two events in a sentence and to elucidate reasons for why sentences containing these connectives can be hard to process. Our findings extend the understanding of young children's connective competence in several important ways. First, we show that at around 3–4 years of age, children perform above chance on these connectives, indicating that they have a basic understanding of these connectives earlier than reported in previous research. Second, we find that between 6 and 7 years of age children are performing at high levels of accuracy, demonstrating the emergence of full competence earlier than has been reported previously (Cain & Nash, 2011; Pyykkönen & Järvikivi, 2012). Third, and critically, we demonstrate that children's competence is substantially modulated by variation in sentence structure and that an independent measure of working memory influences success. These findings indicate that both cognitive and language demands influence the emergence of young children's comprehension of temporal connectives.

The 3‐ to 4‐year‐old children performed above chance but demonstrated fragile understanding of the meanings conveyed by *before* and *after*. We hypothesized that the youngest children's pattern of performance would indicate that they did not have robust knowledge of the temporal relation signaled by the connective (consistent with previous research detailed above) and would rely on a strategy of either order of mention or world knowledge to comprehend sentences. Consistent with this prediction, the pattern of performance for the youngest age group revealed that they relied on an order of mention strategy: The youngest age group was more accurate on chronological order than on reverse order sentences, consistent with some previous research (Clark, 1971; French & Brown, 1977; Johnson, 1975; Trosborg, 1982).

We can turn to the adult literature to understand better the mechanisms underlying this developmental change. In the world around us, we experience events in a chronological order. Therefore, even adult comprehenders appear to expect that language will map onto that experience, displaying processing difficulties when such mapping is violated (Ye et al., 2012; Zwaan & Radvansky, 1998). Therefore, it is likely that children are mapping the events based on their assumption that language maps onto order, rather than focusing on the linguistic information of the connective itself.

In contrast to some previous studies (French & Brown, 1977; Keller‐Cohen, 1987; Trosborg, 1982), there was no evidence that the children relied on world knowledge to process these sentences: Within each age group, children performed comparably whether the order of the two events was supported by world knowledge or not. We believe that our finding for reliance on order of mention rather than world knowledge is robust because the large participant sample size and use of a linguistic sample considerably larger than the norm for this area of research vouchsafed a fair opportunity to observe a world knowledge effect if one were to be found. In addition and critically, our use of a task that minimized cognitive load in producing responses (compared to act‐out tasks; e.g., French & Brown, 1977; Keller‐Cohen, 1987) was designed to ensure maximum sensitivity.

Our findings show that the comprehension performance of 4‐ to 6‐year‐olds was governed by the varying information‐processing demands of different sentence structures (Diessel, 2004; Pyykkönen & Järvikivi, 2012; Ye et al., 2012). Children were more successful in understanding the sequence of events if those events were presented in sentences in chronologically ordered clauses rather than in reverse chronological order, but this order effect was apparent for sentences containing the *before* connective not for sentences containing *after*. This interaction cannot be attributed to a lack of understanding of the connective *before*. Given that it is acquired earlier than *after* (Clark, 1971), it cannot be the case that understanding a *before* sentence is more susceptible to the effect of order because *before* is not understood as well.

As noted in the Introduction, higher demands are made on memory when the sentence elements are presented in reverse chronological order (Ye et al., 2012), when the connective is later acquired (*after*; Clark, 1971; Leech et al., 2001), and when the connective is in the sentence initial position (Diessel, 2004). The difference due to order is revealed only for *before* sentences because the before‐chronological sentences make the lowest demands on memory. They remain easier to comprehend than before‐reverse (initial position, reverse order) sentences consistently throughout the age range in our sample of children. Like the before‐reverse sentences, the after‐chronological (initial position, later acquired connective) and after‐reverse (reverse order, later acquired connective) sentences each possesses two features that tax children's processing capacities. We note that previous studies that have reported adult processing difficulties for reverse chronological sentences have only included order as a factor (Münte et al., 1998; Ye et al., 2012). That is, connective position was held constant and connective type was a confounding variable. Our interest in developmental acquisition motivated us to include connective type in addition to order as a statistical factor, which in turn enabled us to discuss how different connective positions may have influenced findings.

Our findings add to a growing body of research that has reported age‐related differences in children's understanding of temporal connectives (e.g., Clark, 1971). Of notable interest was the high accuracy of responses to before‐chronological sentences that supports our prediction that chronological order, a more familiar connective (*before*), and a medial connective position are factors that allow a word‐by‐word incremental processing strategy (Cain & Nash, 2011; Diessel, 2004; Traxler et al., 1997). Our findings indicate that children as young as 4 years of age process multiple‐clause sentences accurately in this way. The main effect of memory on accuracy indicates that children with higher memory capacities comprehend two‐clause sentences more accurately. Critically, we found that memory could explain why children displayed sentence specific performance. This finding supports previous research that informed us that children's comprehension of two‐clause sentences containing *before* and *after* can be influenced by whether their memory capacity is sufficient to cope with the variability in the processing demands of our sentence structures (Just & Carpenter, 1992; Pyykkönen & Järvikivi, 2012; Ye et al., 2012).

The observed effect of memory suggests that where age effects are found they may, as here, reflect a contribution due to the development of memory capacity. This finding highlights the difficulty of distinguishing the impact of the development of memory from the impact of language development. In addition, our results also highlight the need to study specific connectives within a single temporal class (Cain & Nash, 2011; Crosson & Lesaux, 2013). Our observations suggest that knowledge of *before* was more robust than knowledge of *after*. This may be due to their differences in semantics (Clark, 1971) or to their differing frequency of occurrence as temporal connectives (Leech et al., 2001).

Recent literature suggests that working memory tests that have been used to support capacity‐constrained theories might instead tap into long‐term knowledge of language (Kidd, 2013; Klem et al., 2015). For that reason, we tested whether our proposed memory effects were a result of long‐term knowledge of language by running two models that incorporated vocabulary scores, one with and one without memory. We concluded from model comparisons that vocabulary did not significantly improve the fit compared to equivalent models that did not include vocabulary. The findings confirmed that memory was driving performance. In addition, it is important to note that we manipulated sentence structures while holding vocabulary constant. That is, vocabulary did not vary across experimental conditions (other than for the specific connective itself), which runs counter to this alternative explanation that vocabulary knowledge could be a proxy for the reported memory effects (see also Cain, Patson, & Andrews, 2005, for an example of the separation between vocabulary knowledge and connective comprehension in young children).

We would not dismiss a language account completely. Of note our sentences differed by connective type and we believe that language knowledge variation could explain the general advantage for *before*. In addition, frequency of exposure to a specific sentence structure can influence comprehension (Tomasello, 2003). Therefore, future work should consider both the frequency of our sentence structures and the use of *before* and *after* in parental input and examine whether this maps onto the pattern of development found in the present study.

Convergent with our information processing explanation of children's difficulties with temporal connectives in multiple‐clause sentences, we found that the inclusion of an independent measure of memory improved model fit. However, further evidence is needed to corroborate this account. We used the most sensitive behavioral measure of memory that we could identify for our age groups, but believe that other techniques will support our findings and reveal critical pressure points in the moment‐by‐moment processing of these sentences. The extent to which the factors of event order, connective, and position influence the real‐time processing of connectives in young children may be studied with techniques that do not require a behavioral response, such as using eye tracking within a visual world paradigm (Arnold, Eisenband, Brown‐Schmidt, & Trueswell, 2000) or by recording event‐related potentials to index processing difficulty, as has been done successfully in studies of adults' production of connectives (Habets, Jansma, & Münte, 2008). These techniques would provide fine‐grained measures of processing efficiency and processing cost in critical regions of multiple‐clause sentences (Cozijn, Noordman, & Vonk, 2011). Response times or evoked potential differences for regions where the cognitive demands were greatest, in particular, might be more strongly related to independent measures of memory.

In addition to the limitations discussed earlier, we note that we used experimenter‐constructed sentences, rather than sentences based on natural speech. Previous research on children's understanding of complex sentences shows that difficulties can disappear when target sentences are based on naturalistic speech (Kidd, Brandt, Lieven, & Tomasello, 2007; Rowland & Noble, 2010). However, we contend that these sentences parallel those found in naturalistic speech: Diessel (2004) reports examples of children's and adults' speech (Diessel, 2005, 2008) containing numerous examples of sentences containing temporal connectives that are supported by world knowledge or not, just like our experimental manipulation. Therefore, we do not believe this is the reason for our findings although various pragmatic manipulations could be explored in future research. Furthermore, we did not program the randomization of items to prevent potential priming when the same sentence structure is presented twice in a row. Given the number of items (*N *=* *64), we do not believe that this feature unduly influenced our findings. However, it would be interesting in future work to test if such features could be used to support language comprehension of more difficult syntactic structures, as has been found for language production (e.g., Allen, Haywood, Rajendran, & Branigan, 2011).

A final thought for future research is whether the same factors that influence comprehension of sentences with temporal connectives also influence production. Typically, in terms of language knowledge, comprehension generally precedes production for specific words and grammatical structures (Benedict, 1979; Fraser, Bellugi, & Brown, 1963). One reason for that difference may be the additional planning demands of language production (Diessel, 2004; MacDonald, 2013). However, comprehension and production are related and draw on many of the same cognitive processes (Pickering & Garrod, 2013). There is a clear need for comparison of children's comprehension versus production of complex sentences containing connectives to provide insight into common sources of difficulty. Critically, it would be important to determine whether the processing patterns for specific sentence types reported by the present study map onto performance in a production paradigm.

In summary, the present study demonstrates substantial differences between 3‐ and 7‐year‐old children in their comprehension of two‐clause sentences containing *before* and *after* and the factors that influence performance. The 3‐ to 4‐year‐olds demonstrated poor knowledge of the distinction between the meanings of these two temporal connectives and tended to interpret the event order as the order of mention of events. Older children's performance indicated adequate understanding of these connectives, but the poorer performance of 4‐ to 6‐year‐olds relative to 7‐year‐olds, together with the relations with memory, indicated that comprehension may fail when the processing demands are high. Further research using online measures of sentence processing is required to identify the locus of difficulty when comprehending these sentences, which will help to elucidate the role of processing resources in children's comprehension of multiple‐clause sentences.
